# Association of *ARMS2* genotype with response to anti-vascular endothelial growth factor treatment in polypoidal choroidal vasculopathy

**DOI:** 10.1186/s12886-017-0631-z

**Published:** 2017-12-07

**Authors:** Un Chul Park, Joo Young Shin, Hum Chung, Hyeong Gon Yu

**Affiliations:** 1 Department of Ophthalmology, Seoul National University College of Medicine, 103 Daehak-ro, Jongno-gu, Seoul, 110-799 South Korea; 2 Department of Ophthalmology, Chung-Ang University Hospital, Seoul, South Korea; 3 Retinal Degeneration Research Laboratory, Seoul National University Hospital Biomedical Research Institute, Seoul, South Korea

**Keywords:** Polypoidal choroidal vasculopathy, Anti-vascular endothelial growth factor, Single nucleotide polymorphism, Pharmacogenetics

## Abstract

**Background:**

To investigate whether genetic risk variants for age-related macular degeneration (AMD) are associated with response to intravitreal anti-vascular endothelial growth factor (VEGF) in polypoidal choroidal vasculopathy (PCV) patients.

**Methods:**

This prospective cohort study included 95 treatment-naïve patients that underwent anti-VEGF treatment for PCV for 12 months. Patients were genotyped for 10 single nucleotide polymorphisms in eight AMD-relevant genes. Genotypic association with visual and anatomic outcome measures at 12 months after initial treatment, including mean change in best-corrected visual acuity (BCVA) and total foveal thickness, visual gain of ≥ 15 letters, dry status on optical coherence tomography (OCT), pigment epithelial detachment (PED) regression on OCT, polyp regression on indocyanine green angiography, and injection numbers, were investigated using regression models with adjustment for non-genetic covariates under additive genetic model.

**Results:**

In 81 patients who completed 12-month anti-VEGF monotherapy without photodynamic therapy, significant pharmacogenetic association was found between *ARMS2* rs10490924 and PED regression on OCT. Proportions of PED regression were 26.4% for TT, 45.7% for TG, and 63.6% for GG genotype, showing additive effect of G allele for higher chance of PED regression (OR, 2.96; 95% CI, 1.38–6.36; corrected *P* = 0.043). For entire 95 patients, no significant association was found between candidate polymorphisms and receiving photodynamic therapy within 12 months.

**Conclusions:**

In PCV patients, *ARMS2* rs10490924 showed association with anatomic therapeutic response to anti-VEGF, suggesting pharmacogenetic relationship.

**Electronic supplementary material:**

The online version of this article (10.1186/s12886-017-0631-z) contains supplementary material, which is available to authorized users.

## Background

Polypoidal choroidal vasculopathy (PCV), characterized by inner choroidal vascular networks ending in polypoidal lesions, is generally considered a subtype of choroidal neovascularization (CNV) secondary to age-related macular degeneration (AMD); however, there remains controversy regarding its entity. [1, 2] As an exudative maculopathy resulting in severe vision loss, PCV and neovascular AMD share similar phenotype but PCV often exhibits clinical features distinct from typical neovascular AMD, which suggests a different pathogenesis. For example, they differ in terms of ethnicity, histopathology, natural course, and response to treatment. [2–5]

Currently, the standard treatment for neovascular AMD is the intravitreal injection of vascular endothelial growth factor (VEGF) inhibitors. In pivotal trials, ranibizumab (Lucentis; Novartis, Basel, Switzerland), a recombinant humanized monoclonal antibody that neutralizes all isoforms of VEGF, and aflibercept (Eylea, Bayer HealthCare, Berlin, Germany), a recombinant fusion protein with components of VEGF receptor 1 and 2, have shown significant visual improvements in neovascular AMD patients. [6–8] When used to treat PCV, intravitreal anti-VEGF reduces exudation and hemorrhage effectively, [9–12] although it has limited effect in terms of regressions of polypoidal lesions or branching vascular networks (BVNs). [10–13] In recent multicenter randomized controlled trials, it was reported that visual prognosis after intravitreal ranibizumab for PCV was similar or superior to photodynamic therapy (PDT). [13, 14] Furthermore, PDT-related complications such as subretinal hemorrhage and retinal pigment epithelium (RPE) tear are another reason to prefer anti-VEGF for the treatment of PCV. [15]

Regarding anti-VEGF treatment for neovascular AMD, pharmacogenetic associations have been investigated in many studies to determine whether an individual’s genetic profile might influence therapeutic outcomes. Recent large-scale studies based on well-defined multicenter cohorts of Caucasian neovascular AMD patients found no significant pharmacogenetic association with anti-VEGF response, [16, 17] but it still remains unclear in Asians. [18, 19] But for PCV, which is more common in Asian compared to the Caucasians, only limited number of genetic variants has been tested regarding their influence on therapeutic response to anti-VEGF. [20, 21] The aim of this study was to assess the influence of AMD-relevant genetic variants on therapeutic response to intravitreal anti-VEGF in Korean PCV patients.

## Methods

The treatment protocol and design of this study were approved by the Institutional Review Board of Seoul National University Hospital and were in accord with the tenets of the Declaration of Helsinki. Written informed consent was obtained from all PCV patients at times of treatment and blood sampling for genotyping, and the patients were registered at the retina center of the Seoul National University Hospital (IRB no: 1007–180-325). Patients who started intravitreal anti-VEGF treatment between January 2010 and July 2013 were prospectively recruited. During the study period, the use of anti-VEGF followed a standard treatment protocol of our institute in all patients.

Polypoidal choroidal vasculopathy was diagnosed based on the presence of characteristic polypoidal vascular lesions and BVNs on indocyanine green angiography (ICGA), which was performed using Heidelberg Retina Angiograph system (HRA-spectralis; Heidelberg Engineering, Heidelberg, Germany) equipped with a confocal scanning laser ophthalmoscope. We included macular PCV patients with subfoveal leakage on fluorescein angiogram (FA) and exudative change on OCT who were treated with intravitreal anti-VEGF injections. Additional criteria for inclusion were: (1) an age over 50 years; (2) agreement to genetic analysis; (3) minimum follow-up period of 12 months after the initial treatment. Only one eye per patient was included. Exclusion criteria were: (1) any previous treatment for PCV such as laser photocoagulation, PDT, or anti-VEGF injection; (2) other concomitant ocular disease such as diabetic retinopathy, retinal vascular disease, epiretinal membrane, and high myopia; (3) previous vitreoretinal surgery; and (4) presence of a disciform macular scar or atrophy. No criteria regarding baseline visual acuity were applied.

### Clinical examination and anti-VEGF treatment protocol

At baseline, all patients underwent a complete ophthalmic examination, which included best-corrected visual acuity (BCVA) measurement, slit-lamp examination, intraocular pressure measurement, fundus examination, spectral domain OCT (Cirrus HD-OCT, Carl Zeiss Meditec, Dublin, CA), FA, and ICGA. Visual acuity was measured at each follow-up visit using the Early Treatment Diabetic Retinopathy Study (ETDRS) chart by experienced testers after standardized refraction. The area of abnormal vascular lesion of PCV, including entire BVNs and polypoidal lesions on ICGA, was measured using the HRA software. The spectral domain OCT protocol included 6-mm horizontal and vertical linear scans centered on the fovea and additional scans over the PED to determine the highest point of the PED.

In all patients, anti-VEGF treatment and follow-up schedule were performed according to a standard treatment protocol of our institute, which was comprised of three loading injection followed by as-needed treatment. Initially, patients received a loading dose of three monthly injections of ranibizumab (month 0, 1, 2), and were followed-up monthly. At each visit, ophthalmic examinations including BCVA measurement, fundus examination, and OCT were performed. During follow-up, FA was scheduled to be performed at month 3, but FA or ICGA at month 12 was performed at the physician’s discretion. Anti-VEGF retreatment was administered at monthly visits when any of the following criteria were met: (1) evidence of persistent or new exudative change on OCT; (2) definite increase of pigment epithelial detachment (PED) on OCT as compared with the previous visit; (3) BCVA loss of ≥5 letter from previous visit; and (4) new macular hemorrhage. In some patients whose medical insurance coverage for ranibizumab had been terminated, bevacizumab (Avastin; Roche, Basel, Switzerland) was used for retreatment after discussing the potential risks and benefits of off-label use. Ranibizumab (0.5 mg) or bevacizumab (1.25 mg), both in 0.05 ml of solution, was injected intravitreally under standard sterile conditions. For patients who showed persistent subretinal fluid and PED on OCT despite multiple anti-VEGF injections, a standard PDT as described previously [22] was performed at least 6 months after initial treatment as a rescue therapy. Patients who underwent PDT before month 12 were not included in the primary pharmacogenetic analysis for anti-VEGF responsiveness, but were included in the analysis for association between genetic variants and undergoing PDT.

### DNA preparation and genetic analysis

Approximately 10 mL of peripheral blood was obtained from each patient. A total of 10 candidate polymorphisms in 8 known AMD-associated genes, including *CFH*, *ARMS2*/*HTRA1*, *C2*/*CFB*/*SKIV2L*, *VEGFA*, *PEDF* were genotyped. Genomic DNA was prepared using a nucleic acid isolation device, QuickGene-mini80 (FUJIFILM, Tokyo, Japan). All genetic variants were genotyped using TaqMan SNP genotyping assays (Applied Biosystems Inc.[ABI], Foster City, CA, USA) or SNaPshot Multiplex kit (ABI) according to the manufacturers’ recommendations. Primer sequences of candidate polymorphisms are available on request. The characteristics, genotyping method, and overall genotyping results for candidate polymorphisms are detailed in Table 1.

**Table 1:** Characteristics of candidate genetic markers

Chr	Gene	Location	dbSNP ID	Major/Minor Allele	MAF	Genotyping Method	HWE *P* - value	Genotyping rate
1	*CFH*	I62V	rs800292	G/A	0.290	SNaPshot	0.083	100%
1	*CFH*	Y402H	rs1061170	T/C	0.080	SNaPshot	0.407	100%
6	*C2*	E318D	rs9332739	G/C	0.019	Taqman	0.019	100%
6	*CFB*	R32Q	rs641153	G/A	0.081	SNaPshot	1	98.8%
6	*SKIV2L*	3493G/A	rs429608	G/A	0.088	Taqman	0.463	98.8%
6	*VEGFA*	C-2578A	rs699947	C/A	0.281	SNaPshot	0.782	98.8%
6	*VEGFA*	C936T	rs3025039	C/T	0.247	Taqman	1	100%
10	*ARMS2*	A69S	rs10490924	T/G	0.375	Taqman	0.811	98.8%
10	*HTRA1*	-625A/G	rs11200638	A/G	0.370	Taqman	0.641	100%
17	*PEDF*	Met72Thr	rs1136287	T/C	0.488	Taqman	0.66	100%

*SNP* Single nucleotide polymorphism (dbSNP ID; available at: http://www.ncbi.nlm.nih.gov/SNP/), *MAF* Minor allele frequency, *HWE* Hardy-Weinberg Equilibrium

### Statistical analyses

Assessment of treatment outcome was based on both visual acuity and anatomic features of PCV at month 12. Visual outcome measures were mean BCVA change from baseline and the proportion of patients with visual gain of ≥15 letters. Anatomic outcome measures were mean change in total foveal thickness (TFT) from baseline, no subretinal or intraretinal fluid (dry status) on OCT, PED regression on OCT, and polyp regression on ICGA. With OCT, TFT was measured manually using a caliper provided by the software, and included the retina, subretinal fluid, CNV, and RPE elevation. PED was defined as a focal elevation of the reflective RPE band over an optically clear or moderately reflective space. There was no minimum requirement for PED height, which was measured as the vertical distance from the surface of RPE band to the surface of the choriocapillaris, only if the PED included the area corresponding to the polypoidal lesion on ICGA. Regression of PED was defined as the resolution of sharply elevated focal PED peak on OCT, which reflects the polypoidal lesion of PCV, to the level of adjacent shallow PED and disappearance of the tomographic notch sign. [1, 23] In eyes without a sharp PED peak and notch sign at baseline, a decrease in PED height to less than 50% of baseline was regarded as PED regression. On ICGA, polyps were considered to have regressed when no apparent polypoidal lesions were observed. In addition to visual and anatomic outcome measures, numbers of anti-VEGF injections during 12 months was also evaluated for the association with candidate polymorphisms.

All evaluations for the outcome measures were performed independently by two retinal specialists (UCP and HC) who were unaware of patient personal information, genotypes, and visual outcomes. Measurements for TFT and PED height were averaged, and discrepancies in judgment for dry status and PED regression on OCT, and polyp regression on ICGA were settled by senior investigator (HGY).

The associations between genotypes of candidate polymorphisms and treatment outcome measures at month 12 were evaluated using regression models. For continuous outcome variables including mean changes in BCVA and TFT from baseline and number of injections, a linear regression model was used. For categorical outcome variables, an ordinary logistic regression model was used to calculate odds ratios (ORs) and 95% confidence intervals (CIs). Analyses were performed for each genetic variant independently of other variants with adjustment for non-genetic covariates under additive genetic model. The non-genetic covariates adjusted in the regression analysis were age, sex, smoking status (ever vs. never), and baseline BCVA, TFT, and abnormal vascular lesion area. For the entire patients who completed 12-month follow-up, pharmacogenetic association with undergoing PDT before month 12 were evaluated using logistic regression model. For candidate polymorphisms with a minor allele frequency of <0.1, analysis was also performed using the dominant genetic model.

Statistical analyses were performed using PLINK software version 1.07 (available on http://zzz.bwh.harvard.edu/plink) and SPSS for windows version 21.0 (SPSS Inc., Chicago, IL). Correction for multiple testing was performed using the Bonferroni method. For all statistical tests, corrected *P* values of <0.05 were considered statistically significant.

## Results

Of the 112 patients who met the inclusion/exclusion criteria, two had genetic samples of poor DNA quality, and 15 failed to complete 12-month as-needed regimen of anti-VEGF monotherapy. Three had poor general condition, and 11 were lost to follow-up for unknown cause. One patient quitted anti-VEGF treatment after receiving vitrectomy to repair diffuse hemorrhagic retinal detachment and vitreous hemorrhage which developed at month 9. Of the remaining 95 patients who completed 12-month follow-up, 14 were excluded from primary analysis because they underwent PDT before month 12.

Accordingly, 81 patients (81 eyes) were included for pharmacogenetic analysis. All patients were Korean. Mean age of patients at study entry was 67.6 ± 8.2 years and 49 (60.5%) were male. Mean BCVA improved from 50.6 ± 20.7 letters at baseline to 55.6 ± 25.0 letters at month 12 (*P* = 0.016), and mean TFT of 425.6 ± 199.4 μm at baseline decreased to 344.4 ± 277.2 μm at month 12 (*P* = 0.003). At month 12, 34 patients (42.0%) showed a dry status without any sub- or intraretinal fluid, and 32 patients (39.5%) were considered to have achieved PED regression. In this cohort, genetic influence on polyp regression was not evaluated for entire cohort because ICGA at month 12 was performed at the discretion of physician. Fifty-five patients (67.9%) underwent ICGA at month 12, and 19 patients (34.5%) among them showed regression of polyp on ICGA. The average number of anti-VEGF injections per eye was 5.7 ± 1.9 during 12 months. Ranibizumab was used for 69.5% of all anti-VEGF injections (321/462) and bevacizumab for the remainder. No one cataract surgery during 12-month treatment period. Patient demographics, baseline clinical characteristics, and treatment outcomes at month 12 are presented in Table 2.

**Table 2:** Baseline clinical features and treatment outcomes of the patients

	Total
Number of patients	81
Mean Age, years (range)	67.6 ± 8.2 (50–83)
Male / Female	49 (60.5%) / 32 (39.5%)
Smoking status	
Ever (current / ex-smoker)	32 (39.5%)
Never	49 (60.5%)
Baseline	
Best-corrected visual acuity, ETDRS letters	50.6 ± 20.7
Total foveal thickness, μm	425.6 ± 199.4
Vascular lesion area, mm^2^	3.5 ± 2.1
Treatment outcomes at month 12	
Best-corrected visual acuity, ETDRS letters	55.6 ± 25.0
Visual gain ≥15 letters	25 (30.9%)
Total foveal thickness, μm	344.4 ± 277.2
Dry status on OCT	34 (42.0%)
PED regression on OCT	32 (39.5%)
Polyp regression on ICGA	19 / 55 (34.5%)
Number of injections	5.7 ± 1.9

*ETDRS* Early Treatment Diabetic Retinopathy Study

Potential confounding effect of the non-genetic covariates on outcome measure was evaluated using a multiple linear regression model regarding age, sex, smoking status, and baseline BCVA, TFT, and abnormal vascular lesion area. The regression model revealed that none of the non-genetic covariates had significant effect on BCVA and TFT change from baseline. Regression coefficients (β) with corresponding 95% CIs and *P* values for each non-genetic covariate are presented in Table 3.

**Table 3:** Association of non-genetic covariates with continuous outcome measures

	BCVA change from baseline		TFT change from baseline	
	β (95% CI)	*P* value	β (95% CI)	*P* value
Age	−0.094 (−0.626 to 0.438)	0.725	3.32 (−3.66 to 10.3)	0.346
Sex	6.75 (−4.94 to 50.4)	0.253	−39.8 (−193.1 to 113.5)	0.607
Smoking	5.97 (−5.67 to 17.6)	0.310	−89.1 (−241.7 to 63.6)	0.249
Baseline BCVA	−0.039 (−0.25 to 0.172)	0.714	0.237 (−2.53 to 3.00)	0.865
Baseline TFT	−0.002 (−0.023 to 0.020)	0.885	−0.040 (−0.326 to 0.245)	0.780
Baseline vascular lesion area	−0.368 (−1.09 to 0.348)	0.309	3.99 (−5.40 to 13.4)	0.400

Regression coefficients (β) and *P* values for association of non-genetic covariates with mean change of best-corrected visual acuity and total foveal thickness from baseline are shown

*CI* Confidence interval

### Pharmacogenetic analysis

The overall genotyping rate was 99.5% and less than 2% of data was missing for all genetic variants (Table 1). The distribution of genotypes for each genetic variant was consistent with the HWE (*P* value >0.05), except for *C2* rs9332739. Pharmacogenetic analysis was performed for 10 polymorphisms in 8 genes, and nominal *P* values of <0.005 (= 0.05 / 10) after Bonferroni’s correction for multiple testing were regarded as significant. Regression analyses for pharmacogenetic associations with treatment outcome revealed significant association between PED regression on OCT and genotypes of *ARMS2* rs10490924, while other candidate polymorphism showed no significant association with treatment outcome. Treatment outcome measures at month 12 according to the genotypes of entire candidate polymorphisms are shown in an additional data file [see Additional file 1].

**Fig. 1:**
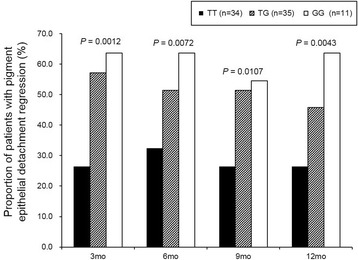
Regression of pigment epithelial detachment according to the *ARMS2* rs10490924 genotypes. The G allele at *ARMS2* rs10490924 had an additive effect on the chance of pigment epithelial detachment regression on optical coherence tomography. Uncorrected *P* values obtained by ordinary logistic regression are shown

At baseline, all 81 patients had PED on OCT which was tomographically corresponding to the polypoidal lesion on baseline ICGA. Proportions of PED regression at month 12 for the TT (major allele homozygote), TG, and GG genotypes of *ARMS2* rs10490924 were 26.4%, 45.7% and 63.6%, respectively. According to the additive genetic model, possession of one more G allele represented a 2.96-times greater chance of achieving PED regression at month 12 (OR, 2.96; 95% CI, 1.38–6.36; uncorrected *P* = 0.0043; corrected *P* = 0.043). Additional assessments for other time points (month 3, 6, and 9) also showed that G allele had an additive effect on the chance of PED regression at all time points, although significant associations were found only at month 3 and 12 (Fig. 1). Baseline characteristics and treatment outcomes according to the genotypes of *ARMS2* rs10490924 are shown in Table 4. Baseline non-genetic covariates did not differ, and treatment outcome measures showed no significant association with *ARMS2* rs10490924 genotypes except the PED regression on OCT.

**Table 4:** Baseline characteristics and treatment outcome measures according to *ARMS2* rs10490924 genotypes

		Genotype			OR (95% CI)*	*P* - value
		TT (34)	TG (35)	GG (11)		
Baseline characteristics	Age	67.0 ± 8.7	68.4 ± 8.1	66.7 ± 7.2	–	0.556^**^
	Sex (male/female)	20 (58.8%)/14	20 (57.1%) / 15	8 (72.7%) / 3	–	0.508^†^
	Smoking (ever/never)	12 (35.3%)/20	15 (42.9%) / 20	5 (45.5%) / 6	–	0.933^†^
	Baseline BCVA, letter	53.1 ± 22.0	45.6 ± 20.8	56.8 ± 12.6	–	0.076^**^
	Baseline TFT, μm	427.1 ± 222.1	452.6 ± 195.6	325.8 ± 102.8	–	0.188^**^
	Baseline PED height	307.3 ± 127.4	299.1 ± 142.2	241.0 ± 132.1		0.203^**^
	Baseline vascular lesion area, mm^2^	6.9 ± 5.4	5.5 ± 4.3	3.2 ± 2.9	–	0.067^**^
Treatment outcome measures (at month 12)	BCVA change from baseline, letter	+0.9 ± 17.7	+9.1 ± 19.5	+5.4 ± 14.3	–	0.338^††^
	TFT change from baseline, μm	−36.8 ± 270.7	−113.3 ± 226.7	−110.1 ± 100.0	–	0.212^††^
	≥ 15 letter gain	8 (23.5%)	13 (37.1%)	4 (36.4%)	1.58 (0.78–3.23)	0.208^††^
	Dry status on OCT	14 (41.2%)	15 (42.9%)	5 (45.5%)	1.19 (0.56–2.53)	0.622^††^
	PED regression on OCT	9 (26.4%)	16 (45.7%)	7 (63.6%)	2.96 (1.38–6.36)	0.004^††^
	Polyp regression on ICGA	8 (32.0%)/25	8 (34.8%)/23	3 (42.9%)/7	1.27 (0.85–1.90)	0.215^††^
	Number of injection	5.7 ± 2.1	5.6 ± 1.7	5.7 ± 1.8	–	0.786^††^

*OR* Odds ratio, *CI* Confidence interval, *BCVA* Best-corrected visual acuity, *TFT* Total foveal thickness, *PED* Pigment epithelial detachment, *OCT* Optical coherence tomography, *ICGA* Indocyanine green angiography

*odds ratio for G allele under additive genetic model

**Kruskal-Wallis test

†Chi-square test

††*P* - value from logistic regression model (categorical outcomes) or linear regression model (continuous outcomes), uncorrected for multiple testing

In 14 patients who underwent PDT because of poor response to anti-VEGF, median time point of PDT was month 8 (range, month 6 to 11). They showed mean BCVA change from baseline of +1.2 ± 9.6 letters at the time of PDT and +4.2 ± 11.7 letters at month 12. Mean TFT change from baseline was −36.6 ± 86.5 μm at the time of PDT and −62.4 ± 134.2 μm at month 12. For 95 patients who completed 12-month follow-up, associations between undergoing PDT before month 12 and candidate polymorphisms were evaluated using logistic regression model, but no significant genetic influence was found after Bonferroni correction (Table 5).

**Table 5:** Genotypic distribution of patients who underwent photodynamic therapy

Gene	Variant	Major allele (M)	Minor allele (m)	Proportion of PDT			OR (95% CI)^*^	*P* – value^**^
				MM	Mm	mm		
*CFH*	rs800292	G	A	9 (20.0%)/45	3 (6.5%)/46	2 (50.0%)/4	0.72 (0.24–2.17)	0.555
*CFH*	rs1061170	T	C	12 (14.8%)/81	2 (15.4%)/13	0 (0.0%)/1	0.56 (0.10–3.02)	0.498
*C2*	rs9332739	G	C	14 (15.1%)/93	0 (0.0%)/1	0 (0.0%)/1	< 0.001 (NA)	0.999
*CFB*	rs641153	G	A	12 (15.2%)/79	1 (7.1%)/14	0 / 0	0.31 (0.03–3.00)	0.312
*SKIV2L*	rs429608	G	A	13 (16.3%)/80	1 (7.7%)/13	0 (0.0%)/1	0.30 (0.03–2.69)	0.283
*VEGFA*	rs699947	C	A	3 (6.7%)/45	8 (20.5%)/39	3 (30.0%)/10	3.02 (1.19–7.68)	0.020
*VEGFA*	rs3025039	C	T	7 (13.2%)/53	6 (16.7%)/36	1 (16.7%)/6	1.22 (0.46–3.24)	0.697
*ARMS2*	rs10490924	T	G	8 (19.0%)/42	5 (12.5%)/40	1 (8.3%)/12	0.40 (0.14–1.15)	0.090
*HTRA1*	rs11200638	A	G	7 (16.7%)/42	6 (14.6%)/41	1 (8.3%)/12	0.48 (0.17–1.33)	0.155
*PEDF*	rs1136287	T	C	3 (13.0%)/23	7 (14.0%)/50	4 (18.2%)/22	1.39 (0.55–3.48)	0.484

Among 95 patients who completed 12-month follow-up, 14 underwent photodynamic therapy and their distributions according to the genotypes of candidate polymorphisms are shown

*PDT* Photodynamic therapy, *MM* Major allele homozygote, *Mm* Heterozygote, *mm* Minor allele homozygote, *OR* Odds ratio, *CI* Confidence interval, *NA* Not applicable

*Odds ratio for minor allele under additive genetic model

**P - value from logistic regression model, uncorrected for multiple testing

## Discussion

In this cohort of Korean PCV patients, we investigated the pharmacogenetic association of therapeutic response to anti-VEGF injection, which included both visual and anatomic outcomes. After 12-month as-needed treatment regimen of anti-VEGF, *ARMS2* polymorphism rs10490924 was found to have significant influence on the anatomic outcome measure. The G allele at rs10490924 had additive effect on the regression of PED on OCT at month 12, but other genetic variants showed no significant association with treatment outcome after anti-VEGF monotherapy.

Despite several clinical features of PCV distinct from typical neovascular AMD, genetic susceptibility to PCV has been investigated in the context of AMD-relevant genes. Major AMD susceptibility genes such as *CFH*, *ARMS2* / *HTRA1*, and *C2* have significant association with PCV. [24] However, genetic association between *ARMS2*/*HTRA1* and PCV is reported to be weaker than its association with AMD, [1, 25–27] and a few genes were found to be specific for PCV. [28, 29] These imply that PCV and typical AMD share molecular mechanism in general, but PCV also has distinct and specific genetic predisposition in part. Similarly, pharmacogenetic association in PCV could differ from typical neovascular AMD, but this has been usually investigated for PDT. To date, *ARMS2* rs10490924, [30] *PEDF* rs12603825, [31] and the *CD36* gene polymorphism rs3173798 [32] have been reported to be associated with visual prognosis of PCV after PDT.

Pharmacogenetic studies on anti-VEGF agents have been conducted in terms of the treatment for neovascular AMD. Although many studies have found significant associations in major AMD-associated genes such as *CFH*, *ARMS2*/*HTRA1*, and *VEGFA*, large-scale pharmacogenetic analyses for participants in multicenter clinical trials such as the CATT or IVAN, most of which were Caucasian neovascular AMD patients, found no significant association between the studied genetic variants and anti-VEGF treatment outcomes. [16, 17] In our previous study, which included 394 Korean patients with typical neovascular AMD but excluded PCV based on ICGA finding, we found that *VEGFA* and *ARMS2*/*HTRA1* polymorphisms are associated with long-term anti-VEGF treatment outcome. [18] However, most of anti-VEGF pharmacogenetic studies for neovascular AMD did not describe whether PCV was considered as an inclusion or exclusion criterion, and one cannot rule out the possibility that some PCV patients might have been included. As far as we know, only two studies have reported pharmacogenetic association of anti-VEGF separately for PCV as a subgroup analysis, but sample sizes were small. [20, 21] Hata et al. [20] reported that *ARMS2* rs10490924 was associated with the 12-month visual acuity but not with 24-month in PCV patients treated with ranibizumab, but Yamashiro et al. [21] reported no association of *CFH* and *ARMS2* genes with visual prognosis.

Among the treatment outcome measures evaluated in this study, only PED regression was found to be associated with genotypes of candidate polymorphisms. The additive genetic model showed that G allele at *ARMS2* rs10490924 significantly increases the chance of PED regression, which suggests that *ARMS2* polymorphism has influence on the regression of vascular lesion below RPE after anti-VEGF treatment. In addition, although insignificant, other treatment outcome measures such as BCVA change and TFT change from baseline showed greater results in patients with G allele (TG and GG genotypes) at rs10490924, supporting the relevance of this polymorphism to the therapeutic response to anti-VEGF in PCV. In a recent study, PCV patients with TT or TG genotypes of rs10490924 had higher risks of recurrence and visual acuity deterioration compared to GG genotype during the second year of combined treatment of PDT and ranibizumab. [33] Considering that GG genotype showed greater macular thickness reduction than other genotypes in our previous pharmacogenetic study for typical neovascular AMD, [18] it appears that *ARMS2* might be a common genetic factor for anatomic response to anti-VEGF for both PCV and typical neovascular AMD. However, role of *ARMS2* gene polymorphism in the therapeutic response to anti-VEGF is still unclear because ARMS2 protein has been poorly characterized and its function is still unknown. [34]

In this study, baseline PED height was lowest in patients with GG genotype at rs10490924. Also, patients with T allele (TT and TG) showed greater baseline TFT, vascular lesion area and worse baseline BCVA suggesting worse influence of T allele at rs10490924 on PCV phenotype. Although the differences did not reach statistical significance, this seems to be due to small sample size of the present study. Previously, TT genotype at rs10490924 was reported to be associated with a severe PCV phenotype with larger lesion size or poorer visual acuity. [27, 35] Baseline clinical heterogeneity among genotypes should be considered when interpreting significant association with treatment outcome, because different severity of PCV at baseline might have affected treatment outcomes and would be translated into different anti-VEGF response. However, it is also possible that *ARMS2* genotype may affect both clinical severity and anti-VEGF response in PCV.

One of the most characteristic OCT findings of PCV is a notching sign caused by a sharply elevated PED with or without connecting lower PED, which is thought to be caused by a polypoidal lesion and adjacent BVNs. [1] A recent study on qualitative analysis of the tomographic features of PCV showed that spectral domain OCT can provide a useful alternative to ICGA for the detection of PCV with high sensitivity and specificity. [36] Repeatability of ICGA is limited due to its invasiveness, and anatomic improvement of vascular lesion of PCV in this study were assessed based on the resolution of sharply elevated PED peak on spectral domain OCT. However, not all PCV patients show this pattern of PED on OCT, and we adopted the decrease in PED height as the criteria for PED regression in those patients. Considering that PED height decreases 33 to 50% from baseline after anti-VEGF, [37, 38] ≥ 50% decrease seemed to represent above average response. [39] Unlike PED regression on OCT, polyp regression on ICGA did not show any significant genetic association in this study. The ICGA at month 12 was not performed when patients were reluctant to have angiography and PCV activity could be judged clearly based on fundus and OCT finding. Only a part of PCV patients could be evaluated for the pharmacogenetic effect on polyp regression, and this could be the cause for this discrepancy. In addition, it is possible that PED regression on OCT may not actually reflect the regression of polyp on ICGA.

In this study, 14 patients who were excluded from analysis because of receiving PDT before month 12 represent poor responders to anti-VEGF monotherapy. The influence of genetic variants on the poor response necessitating PDT was evaluated for 95 patients who completed 12-month follow-up, but significant association was not found. To assess the influence of poor responders’ exclusion, we performed additional pharmacogenetic analysis for 95 patients, including 14 who underwent PDT, entering their treatment outcome measures at the time of PDT into the analysis of month 12 results (last observation carried forward method) but found equivalent results to the analysis for 81 patients. Under same statistical analyses, *ARMS2* rs10490924 showed significant association with PED regression on OCT with beneficial effect of G allele (data not shown), while other variants showed no association.

This study has several limitations. First, the number of patients included was small, and results should be interpreted cautiously due to limited statistical power. In addition, it might be impossible to control all the potential confounding factors and collinearities due to small sample size. Second, the influence of patients who were lost before month 12 was not assessed. These patients quitted in the early course of treatment, and their long-term outcome is difficult to evaluate based on early results considering that PCV recurrence during follow-up could change the clinical course. Third, recently found PCV-specific genes such as cholesteryl ester transfer protein were not included as candidate polymorphisms. [28, 29] Fourth, change of polypoidal lesion as determined by ICGA was not evaluated for entire cohort. On the other hand, this study is strengthened by the use of a standardized anti-VEGF treatment protocol of three loading dose followed by as-needed injection and the recruitment of treatment-naïve PCV patients at a single center, which would have minimized the possible bias from different treatment regimen. In addition, treatment response was assessed multilaterally using visual and anatomic parameters.

## Conclusions

In conclusion, the *ARMS2* rs10490924, one of major AMD susceptibility loci, seems to be associated with anatomic outcomes after anti-VEGF monotherapy in Korean PCV patients. Despite the limited statistical power due to small sample size, our result suggests that genetic background of a PCV patient might influence therapeutic response to anti-VEGF. We suggest further investigations be conducted with larger samples, longer follow-up period, and a wide range of candidate variants.

## Additional file

Additional file 1: This file includes additional table which includes treatment outcome measures according to the genotypes of entire candidate polymorphisms. (DOCX 24 kb)

## Abbreviations

AMD: age-related macular degeneration
BCVA: best-corrected visual acuity
BVN: branching vascular networks
CNV: choroidal neovascularization
ETDRS: Early Treatment Diabetic Retinopathy Study
FA: fluorescein angiogram
ICGA: indocyanine green angiography
OR: odds ratio
PCV: Polypoidal choroidal vasculopathy
PDT: photodynamic therapy
PED: pigment epithelial detachment
RPE: retinal pigment epithelium
TFT: total foveal thickness
VEGF: vascular endothelial growth factor
