# Supplementary material for: Association of ARMS2 genotype with response to anti-vascular endothelial growth factor treatment in polypoidal choroidal vasculopathy
Source: BMC Ophthalmol. 2017 Dec 7;17:241. doi: 10.1186/s12886-017-0631-z (PMC5719580; doi:10.1186/s12886-017-0631-z)
Supplement: Additional file 1: — This file includes additional table which includes treatment outcome measures according to the genotypes of entire candidate polymorphisms. (DOCX 24 kb) [file 12886_2017_631_MOESM1_ESM.docx]

**Additional file 1: Table S1.** Treatment outcome measures according to the genotypes of candidate polymorphisms.

| **Genetic variants** | **Genotypes**  **(number)** | **Treatment outcome measures** | | | | | | |
| --- | --- | --- | --- | --- | --- | --- | --- | --- |
|  |  | **BCVA change (letter)** | **TFT change (µm)** | **≥ 15 letter gain** | **Dry status on OCT** | **PED regression on OCT** | **Polyp regression on ICGA** | **Number of injection** |
| ***CFH* rs800292** | **GG (36)** | +0.7 ± 19.9 | -52.9 ± 262.4 | 8 (22.2%) | 21 (58.3%) | 12 (33.3%) | 9 (36.0%) / 25 | 5.9 ± 2.0 |
|  | **GA (43)** | +7.2 ± 16.0 | -97.6 ± 220.7  8 | 16 (37.2%) | 23 (53.5%) | 18 (41.9%) | 10 (34.5%) / 29 | 5.5 ± 1.8 |
|  | **AA (2)** | +16.5 ± 19.1 | -238.7 ± 146.0 | 1 (50.0%) | 1 (50.0%) | 2 (100.0%) | 0 (0.0%) / 1 | 4.2 ± 1.4 |
|  | ***P* - value^*^** | 0.039 | 0.255 | 0.207 | 0.611 | 0.079 | 0.795 | 0.075 |
| ***CFH* rs1061170** | **TT (69)** | +6.1 ± 18.4 | -98.2 ± 239.1 | 24 (34.8%) | 41 (59.4%) | 31 (44.9%) | 16 (34.0%) /47 | 5.6 ± 1.8 |
|  | **TC (11)** | +3.1 ± 10.4 | +33.4 ± 225.1 | 1 (9.1%) | 4 (36.4%) | 0 (0%) | 3 (37.5%) / 8 | 6.3 ± 2.1 |
|  | **CC (1)** | -42 | -171.9 | 0 (0.0%) | 0 (0.0%) | 1 (100.0%) | 0 / 0 | 3 |
|  | ***P* - value^*^** | 0.043 | 0.551 | 0.092 | 0.237 | 0.133 | 0.742 | 0.914 |
| ***C2* rs9332739** | **GG (79)** | +5.2 ± 18.2 | -74.5 ± 231.3 | 24 (30.4%) | 45 (57.0%) | 31 (39.2%) | 19 (35.2%) / 54 | 5.6 ± 1.9 |
|  | **GC (1)** | +21 | - 699.2 | 1 (100.0%) | 0 (0.0%) | 1 (100.0%) | 0 / 0 | 8 |
|  | **CC (1)** | -15 | +6.5 | 0 (0.0%) | 0 (0.0%) | 0 (0.0 %) | 0 (0.0%) / 1 | 5 |
|  | ***P* - value^*^** | 0.415 | 0.613 | 0.793 | 1.000 | 0.841 | 1.000 | 0.905 |
| ***CFB* rs641153** | **GG (67)** | +5.4 ± 19.2 | -77.2 ± 258.2 | 22 (32.8%) | 38 (56.7%) | 28 (41.8%) | 17 (35.4%) / 48 | 5.7 ± 1.8 |
|  | **GA (13)** | +4.5 ± 13.2 | -107.5 ± 112.2 | 3 (23.1%) | 7 (53.8%) | 4 (30.8%) | 2 (28.6%) / 7 | 5.5 ± 2.1 |
|  | **AA (0)** | NA | NA | NA | NA | NA | 0 / 0 | NA |
|  | ***P* - value^*^** | 0.835 | 0.592 | 0.680 | 0.951 | 0.504 | 0.541 | 0.807 |
| ***SKIV2L rs429608*** | **GG (67)** | +5.2 ± 19.0 | -68.6 ± 246.4 | 21 (31.3%) | 38 (56.7%) | 27 (40.3%) | 17 (35.4%) / 48 | 5.7 ± 1.8 |
|  | **GA (12)** | +6.6 ± 14.3 | -144.0 ± 204.6 | 4 (33.3%) | 6 (50.0%) | 4 (33.3%) | 2 (28.6%) / 7 | 5.9 ± 2.3 |
|  | **AA (1)** | -15 | +6.5 | 0 (0.0%) | 0 (0.0%) | 0 (0%) | 0 / 0 | 5 |
|  | ***P* - value^*^** | 0.903 | 0.455 | 0.818 | 0.622 | 0.571 | 0.541 | 0.971 |
| ***VEGFA* rs699947** | **CC (42)** | +3.7 ±15.3 | -82.4 ± 172.3 | 8 (19.0%) | 25 (59.5%) | 16 (38.1%) | 10 (34.5%) / 29 | 5.8 ± 1.9 |
|  | **CA (31)** | +4.0 ± 21.6 | -65.9 ± 325.1 | 12 (38.7%) | 15 (48.4%) | 12 (38.7%) | 7 (33.3%) /21 | 5.7 ± 1.9 |
|  | **AA (7)** | +15.3 ± 15.7 | -88.0 ± 68.2 | 4 (57.1%) | 4 (57.1%) | 3 (42.9%) | 2 (40.0%) / 5 | 5.1 ± 1.2 |
|  | ***P* - value^*^** | 0.327 | 0.868 | 0.057 | 0.723 | 0.735 | 0.325 | 0.302 |
| ***VEGFA* rs3025039** | **CC (46)** | +3.8 ± 15.3 | -100.2 ± 221.4 | 11 (23.9%) | 27 (58.7%) | 17 (37.0%) | 11 (34.4%) / 32 | 6.0 ± 1.8 |
|  | **CT (30)** | +6.6 ± 22.7 | -37.5 ± 274.2 | 13 (43.3%) | 14 (46.7%) | 10 (33.3%) | 7 (35.0%) / 20 | 5.5 ± 1.8 |
|  | **TT (5)** | +9.9 ± 11.6 | -169.5 ± 129.6 | 1 (20.0%) | 4 (80.0%) | 5 (100.0%) | 1 (33.3%) / 3 | 3.6 ± 0.5 |
|  | ***P* - value^*^** | 0.327 | 0.882 | 0.325 | 0.632 | 0.108 | 0.783 | 0.021 |
| ***ARMS2* rs10490924** | **TT (34)** | +0.9 ± 17.7 | -36.8 ± 270.7 | 8 (23.5%) | 14 (41.2%) | 9 (26.4%) | 8 (32.0%) / 25 | 5.7 ± 2.1 |
|  | **TG (35)** | +9.1 ± 19.5 | -113.3 ± 226.7 | 13 (37.1%) | 15 (42.9%) | 16 (45.7%) | 8 (34.8%) / 23 | 5.6 ± 1.7 |
|  | **GG (11)** | +5.4 ± 14.3 | -110.1 ± 100.0 | 4 (36.4%) | 5 (45.5%) | 7 (63.6%) | 3 (42.9%) / 7 | 5.7 ± 1.8 |
|  | ***P* - value^*^** | 0.338 | 0.212 | 0.208 | 0.622 | 0.004 | 0.215 | 0.786 |
| ***HTRA1* rs11200638** | **AA (35)** | +1.9 ± 17.7 | -36.2 ± 275.5 | 8 (22.9%) | 15 (42.9%) | 10 (28.6%) | 8 (32.0%) / 25 | 5.7 ± 2.1 |
|  | **AG (35)** | +7.6 ± 19.8 | -117.1 ± 227.5 | 13 (37.1%) | 15 (42.9%) | 15 (42.9%) | 8 (34.8%) / 23 | 5.6 ± 1.7 |
|  | **GG (11)** | +4.4 ± 14.1 | -110.1 ± 100.0 | 4 (36.4%) | 5 (45.5%) | 7 (63.6%) | 3 (42.9%) / 7 | 5.7 ± 1.8 |
|  | ***P* - value^*^** | 0.615 | 0.276 | 0.171 | 0.679 | 0.014 | 0.215 | 0.692 |
| ***PEDF* rs1136287** | **TT (20)** | +2.9 ± 19.0 | -54.6 ± 144.5 | 6 (30.0%) | 10 (50.0%) | 7 (35.0%) | 5 (35.7%) / 14 | 6.2 ± 2.1 |
|  | **TC (43)** | +7.0 ± 16.9 | -112.6 ± 245.4 | 14 (32.6%) | 23 (53.5%) | 16 (37.2%) | 9 (32.1%) / 28 | 5.9 ± 1.7 |
|  | **CC (18)** | +3.1 ± 20.6 | -35.9 ± 301.6 | 5 (27.8%) | 12 (66.7%) | 9 (50.0%) | 5 (38.5%) / 13 | 4.6 ± 1.6 |
|  | ***P* - value^*^** | 0.877 | 0.891 | 0.920 | 0.748 | 0.385 | 0.478 | 0.024 |

*: *P* - value from logistic regression model (categorical outcomes) or linear regression model (continuous outcomes), uncorrected for multiple testing
